# Clinical and genetic characterization of basal cell carcinoma and breast cancer in a single patient

**DOI:** 10.1186/2193-1801-3-454

**Published:** 2014-08-22

**Authors:** Alessandra Morelle, Rodrigo Cericatto, Ana Cristina Victorino Krepischi, Itamar Romano Garcia Ruiz

**Affiliations:** Moinhos de Vento Hospital, Porto Alegre, RS Brazil; International Center for Research and Training, A. C. Camargo Cancer Hospital/National Institute of Science and Technology in Oncogenomics; Biosciences Institute, Genetics and Evolutionary Biology, Sao Paulo, Brazil; Dermatology Department, Medical Investigation Laboratory (LIM 56), School of Medicine, University of Sao Paulo, Sao Paulo, Brazil

**Keywords:** Invasive ductal breast carcinoma, Basal cell carcinoma, Array-CGH, Copy number alterations

## Abstract

**Introduction:**

Multiple environmental and genetic factors are involved with the development of basal cell carcinomas (BCC), as well as with breast cancers. Tumor initiation and progression are often associated with genomic instability such as aneuploidies, and gains or losses of large chromosomal segments, known as copy number alterations (CNAs). CNAs have been successfully detected using the microarray comparative genomic hybridization technique (array-CGH) at high resolution. Data thus obtained are useful to identify specific genomic aberrations, to classify tumor stages, and to stratify subgroups of patients with different prognosis and clinical behaviors.

**Case description:**

Clinical study of a 66-year-old white female identified two primary tumors, a ductal invasive grade-II carcinoma of the breast, and one nodular BCC. Germline and tumor genomic survey utilized the 180 K array-CGH analysis to investigate chromosomal alterations.

**Discussion and evaluation:**

Several chromosomal anomalies were detected in the breast tumor genome, including focal ~422 Kb 13q13.3 microdeletion. In the BCC, amplification of a chromosome 6 spanning the centromere region between the cytobands 6p23 and 6q12 was identified. Several 6p amplified genes correspond to families of histone and human leukocyte antigen genes, whereas some of the CNAs found in the breast tumor are uncommon. No germline CNA was detected in the normal skin of the patient at this technical resolution.

**Conclusion:**

CNAs found in the two different tumors of the patient constitute independent events arisen in the somatic lineage. Relevant genes to both carcinogenesis and progression are to be affected by these CNAs.

**Electronic supplementary material:**

The online version of this article (doi:10.1186/2193-1801-3-454) contains supplementary material, which is available to authorized users.

## Introduction

Cancer establishment depends on multistep alterations of the genome including gene mutations, deletions or insertions, and epigenetics aberrations, leading to decreased apoptosis, increased cell proliferation, angiogenesis, invasion and metastasis. The tumor progression is often associated with gross genomic instability such as aneuploidies, and gains and losses of chromosomal regions.

Copy number alterations (CNAs) of DNA segments have been successfully detected by the array-CGH technique at high resolutions allowing the simultaneous investigations of thousands of genes in the entire genome. Amplification and homozygous deletions, detected by array-CGH may indicate candidate oncogenes, or cancer suppressor genes, respectively.

### Clinical and molecular aspects

Although BCC presumably develops from epidermal stem cells of the outer root sheet of the hair follicle, the precise origin of BCC is still unknown. Apart from the environmental exposure and immunosuppressive therapy, people with a fair skin type-I complexion including red or blonde hair, light colored eyes, freckling, and those with a history of intermittent sun exposure, especially ultraviolet B radiation and severe sunburn during childhood, are at highest risk (Göppner and Leverkus 2011).

The estimated number of new-cases of non-melanoma skin cancer in Brazil was approximately 160,000 in 2012, the risk being 75 and 84 new cases per 100,000 for males and females, respectively. Rates vary across the country according to specific regions and population ethnical composition, and are particularly high in the Central, South and Southern Brazilian regions (INCA 2012).

BCCs are classified into five types: nodular-ulcerative, pigmented, sclerodeiform or fibrosing, superficial and fibroepithelioma. The nodular ulcerative is the most common form, usually isolated and affecting mainly the head and neck (Chinem and Miot 2011). Less than 1% BCC metastasizes, mostly to lymph nodes, lungs and bones (Bader and Scarborough 2000). Treatment options are focused on local control, including surgical techniques as curettage and electrodessication, cryosurgery, surgical excision, and Mohs micrographic surgery. Nonsurgical approaches include radiotherapy, topical injection therapy, and photodynamic therapy (Rubin et al. 2005).

Mutations in genes associated with the sonic hedgehog (SHH) signaling pathway, as well as defects in repair genes or up-regulation of transcription factors have been implied in the development of BCC (Iwasaki et al. 2013). Also, several genes involved in oxidative phosphorylation and energy metabolism are up-regulated in both melanoma and BCC (Xu et al. 2012).

Germline deletions affecting the *PTCH1* gene is responsible for the Gorlin syndrome phenotype also known as Nevoid Basal Cell Carcinoma Syndrome (NBCCS). A Gorlin Syndrome patient bearing 46,XY and a *de novo* del(9)(q22.3q31.3) was reported (Chen et al. 2006). This deletion that was detected on array-CGH analysis besides other techniques, led to the heterozygous loss of the *PTCH1* gene (9q22.3). Another case also showed deletion on the 9q arm [46,XX,del(9)(q22.1q22.32)], including the *PTCH1* and *ROR2* genes: A 12 year old girl exhibited several NBCCS features but no tumors yet, since BCCs usually manifest after puberty (Nowakowska et al. 2007). Similarly, a 46,XY patient who showed abnormal non-Gorlin features in early age, and exhibited a *de novo* deletion in this same region encompassing the *PTCH1* gene was described (de Ravel et al. 2009). Recent study on basaloid squamous cell carcinomas (SCC) and carcinosarcoma, which are set apart from classical SCC, detected CNAs gains in chromosomes 2p, 7q, 11q, and losses at 13q31ter (Schaefer et al. 2011).

Regarding breast cancer, about one million new cases per year are referred worldwide, from which 35% patients will eventually die. In Brazil, the estimative of new breast cancer cases in 2012 was 52,680 and 12,705 deaths (INCA 2012). Breast cancer is a heterogeneous disease with histological differences within tumors and between patients. The cancer development in the normal mammary gland depends on stem cells, neighboring cells, including fat cells and fibroblasts that play distinct roles through specific signaling pathways (Ercan et al. 2011).

Several factors have been associated with an increased risk of breast cancer, such as family history, nulliparity, early menarche, advanced age, and a personal history of in situ or invasive breast cancer. Of all women with breast cancer, 5 to 10% may have a germline mutation of the genes *BRCA1* and *BRCA2* (Blackwood and Weber 1998). The estimated lifetime risk of developing breast cancer for women with *BRCA1* and *BRCA2* mutations is 40% to 85% (Frank et al. 1998).

The treatment of breast cancer includes surgery, radiation therapy, chemotherapy, and hormone therapy. The prognosis and selection of therapy may be influenced by clinical and pathological features, such as age, menopausal status, stage disease, histological and nuclear grade, hormone receptor status, over expression of human epidermal growth factor 2 (HER2/neu) and proliferative capacity of the tumor (Simpson et al. 2000). Molecular profiling has led to the classification of breast cancer into five distinct subtypes: basal-like, HER2+, normal, luminal A and luminal B (Perou et al. 2000; Sørlie et al. 2001). Pathological and molecular markers as well as gene expression profiles are useful to estimate the risk of breast cancer recurrence after surgery.

The array-CGH technology has been used to identify subgroups of patients with different prognosis and clinical behaviors, as well as to discover susceptibility genes, oncogenes and tumor suppressor genes. Distinct array-CGH profiles were described for ductal and lobular, tubular/tubulo lobular, medullary, micropapillary and secretory breast carcinomas. Loss of 16q has been associated with good prognosis, being more common in lobular than ductal carcinomas. Chromosome 8p11-12 (about 10 Mb) is a gene-dense region that has been implicated in various tumor types. *BRCA1*-mutated, *BRCA2*-mutated, and sporadic breast tumor classes presented loss of chromosome arm 8p, and gain of arm 8q (Didraga et al. 2011). Amplification of 8p11-12, 11q13-14, and 20q13 was correlated with poor prognosis (Climent et al. 2007).

Twenty-three female patients bearing breast cancer, eleven of them with more than one primary tumor type, were studied (Silva et al. 2012). Four out of the eleven breast cancer patients beard a skin cancer. The *BRCA1, BRCA2* and *TP53* genes studied by the MLPA technique showed no CNAs in the germline (blood DNA), except for a single breast cancer patient. A micro deletion flanked by repetitive *Alu* sequences in the *BRCA1* gene at 17q21.31 was detected by array-CGH profile and sequencing.

In the present report, a BCC and a breast carcinoma, developed simultaneously in two different areas of the skin of a patient, were clinically and genetically characterized. The array-CGH analysis detected different somatic CNAs in both tumors, but not in the normal tissue.

## Material

The clinical diagnosis and history of the 66 year old female patient comprise an ulcerative lesion on the left dorsum that evolved within about 3 years. Another ulcerative lesion developed on the right breast within about 2 years. The menarche and menopause were attained when the patient was 13 and 55 year old, respectively. Hormonal replacement was carried out for more than 10 years. The patient’s sister died due to cutaneous melanoma.

## Method

Genomic DNA was isolated from fresh frozen samples of the breast cancer and the BCC, labeled with fluorescence and cohybridized with normal reference DNA. Microarray-Comparative Genome Hybridization analysis was performed as previously described (Krepischi et al. 2012) using a whole-genome 180 K platform (Agilent Technologies, Santa Clara, California, USA), containing about 180,000 oligonucleotides throughout the human genome, according to the manufacturer’s instructions. A gain or loss in copy number was considered when the log_2_ ratio of the Cy3/Cy5 intensities of a given genomic segment was >0.3 or < -0.3, respectively. A commercially available pool of female DNAs (Promega, Madison, WI, USA) was used as the reference DNA in the array-CGH experiments.

## Results and discussion

### Clinical analysis

The mammography revealed a speculated nodule with high radio sensitivity, associated to skin retraction, at the upper internal right breast. The echography showed a 2.6 cm tumor that reached the skin and pectoral muscle. Adjacent to this nodule, another one was identified within 1.4 cm from the former, which exhibited similar characteristics and measured 2.0 cm, attaining also the pectoral muscle.

**Figure 1:**
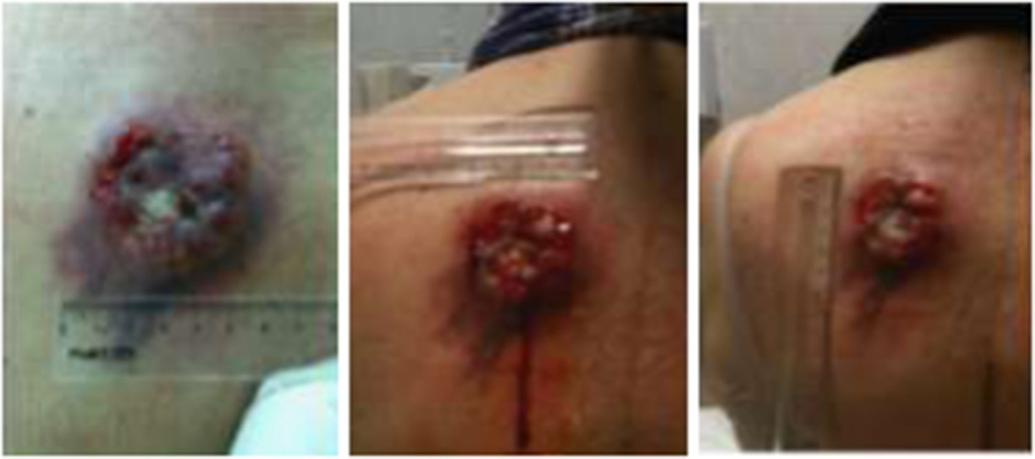
**The ulcerative lesion developed on the left dorsum was further identified in the biopsy as an adenoid BCC.**

**Figure 2:**
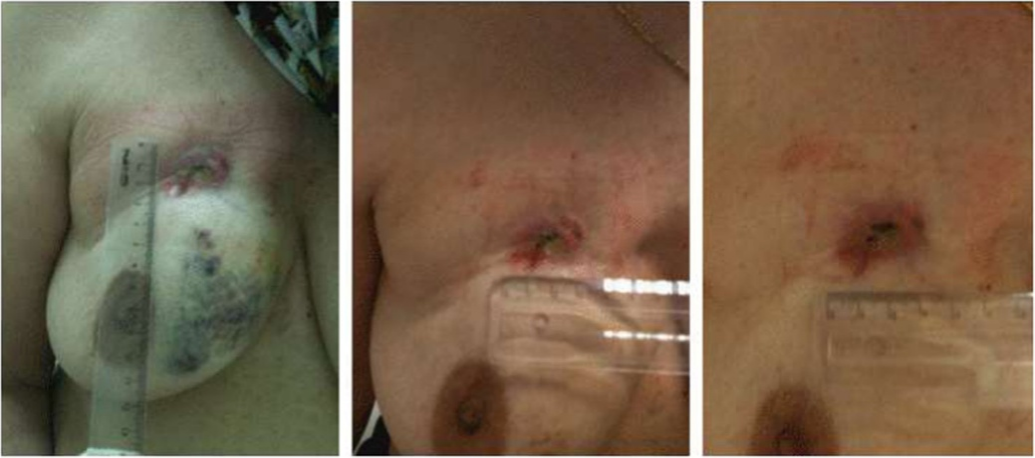
**Ductal invasion carcinoma at the right breast is compatible with a primary breast cancer.** The tumor with histological degree II, showed accentuated formation of tubules, moderate mitotic index, macroscopic metastasis in lymph nodes, angio lymphatic invasion and infiltration of the skin.

Biopsies revealed an adenoid BCC lesion at the left dorsum (Figure 1); and a ductal invasor carcinoma (histological degree II; accentuated formation of tubules; moderate mitotic index; macroscopic metastasis in lymph nodes; and angio lymphatic invasion) at the right breast that infiltrated the skin, compatible with a primary breast cancer (Figure 2). The patient was submitted to neoadjuvant chemotherapy protocol AC and both lesions were surgically resected. Further, adjuvant radiotherapy and electrons beam were used at the left dorsum. The patient started the adjuvant treatment for the breast carcinoma with hormonal blocking via oral anastrosole 1 mg/day, and till now has no evidence of neoplasia activity.

### Molecular array-CGH profile in BCC

**Figure 3:**
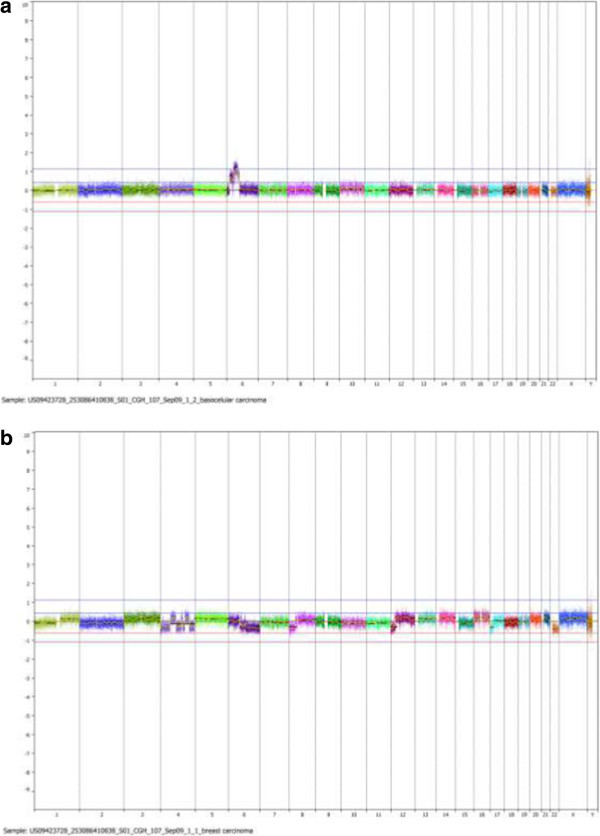
**CNAs in whole BCC and breast cancer genomes are shown. a**. The single amplification found was located at chromosome 6p of the BCC sample. **b**. Several chromosomes were attained by deletions in the breast carcinoma.

A genomic gain in the BCC sample affected about 53 Mb of the chromosome 6 mapped to 6p23–q12, at chr6: 14838756–68055170 (Genome Build Hg18) (Figure 3; Additional file 1: Figure S1). The affected region exhibit distinct levels of gain, one of them compatible with a moderate amplification. Many genes are found in this region (Additional file 2: Table S1), some of them already associated with cancer, such as the tumor suppressor gene *CDKN1A*, at 6p21.2.

The amplifications found at 6p21.3 attained particularly the histone cluster 1 H1d gene family (*HIST1H1D*) responsible for chromosome structure and function, and the great number of genes encoding several classes of the major histocompatibility complex antigens (*HLA*). There are 173 genes at the end of the 6p23-p11 which are repeated in the 6p21.2-p11.2. This pericentromeric gain in the chromosome 6 suggests that the gain/amplification may be carried by the tumor genome as a ring chromosome containing the affected sequence.

The specific gene *PTCH1* was also investigated, but no alterations were found at the resolution used (~4 Kb).

Although the chromosome 6 is often deleted in skin cancers, array-CGH studies have also shown chromosome 6 gains in SCC (Li et al. 2010; Li et al. 2012), melanoma (Pirker et al. 2010) and Merckel carcinoma (Paulson et al. 2009). Small supernumerary marker chromosomes and also ring chromosomes have been found in several instances as derived from chromosome 6 (Huang et al. 2012; Guilherme et al. 2012). Deletions at the end of both arms of one allele 6 are often involved in this rare *de novo* event. The presence of a large amount of euchromatin extending beyond distal 6p12.1 and proximal 6q12 (ring chromosome) has been associated with an abnormal phenotype on a girl 46,XX,r(6)(p25q27) (Ciocca et al. 2013).

### Molecular array-CGH profile in breast carcinoma

Contrarily to the genome alterations observed in the BCC, which were restricted to 6p, a great number of CNAs affecting several chromosomes (Figure 3; Additional file 3: Figure S2) and genes (Additional file 2: Table S1) were identified in the breast carcinoma. The alterations include a total loss of whole chromosome 22, and partial aneuploidy of large chromosomal regions at 4p, 4q, 6q, and 17p, some exhibiting a complex genomic pattern. A submicroscopic focal deletion ~422 Kb was found at 13q13.3 and affected only three genes: *LINC00547* (long intergenic non-coding RNA 547); *TRPC4* (transient receptor potential cation channel, subfamily C, member 4); and *POSTN*. The *POSTN* gene encodes the osteoblast specific factor periostin that is associated with cellular adhesion; it is expressed in normal breast tissue, and is up regulated in breast cancer (Zhang et al. 2010).

Specific *BRCA*1/*BRCA2* genes were also investigated, but no alterations were detected*.*

Breast cancer tissues often show alterations in chromosomes 13q and/or 22 (Climent et al. 2007; Li et al. 2009; Li et al. 2010; Didraga et al. 2011). Among the array-CGH imbalances detected in 75% invasive ductal carcinoma breast cancer samples, there was chromosomal loss of 13q in 3 cases with lymph node metastasis, besides chromosomal gains of +1q, +17q, and +8q (Ghaffari et al. 2008). Loss of the 13q14 region was also detected in benign mammary and vaginal myofibroblastomas (Magro et al. 2012). Deletions detected at 13q and 14q were proposed as hallmarks of *BRCA2*–mutant tumors (Rouault et al. 2012). Besides being the *BRCA2* gene locus (13q12), the 13q region harbors the tumor suppressor gene *RB1* and the gene encoding the transcription factor *FOX01*.

The 17p arm harbors among several other genes, the *TP53* tumor suppressor gene and *HIC1* (zinc-finger protein hypermethylated in cancer 1) gene, at 17p13.1 and 17p13.3, respectively, that were deleted in the present breast tumor sample.

Losses in chromosome 22 ranged from the whole 22q to the gain of just a few hundred kb in 11% of breast cancers, besides several imbalances present in other samples (Benetkiewicz et al. 2006). A candidate tumor suppressor gene *MYO18B* was located in this region (Yanaihara et al. 2004). One subgroup of breast tumors without known *BRCA1* and *BRCA2* mutations showed specific gain of the 22 chromosome (Didraga et al. 2011). Similarly to the findings in the present study, loss of 4q, 6q, and 13q were detected.

No deletions or duplications >300 kb were identified in the normal tissue of the patient. Specific investigation concerning *BRCA1* (at ~20 Kb resolution), *BRCA2* and *PTCH1* (at ~4 Kb resolution) genes did not detect submicroscopic duplications or deletions in the control sample.

## Conclusion

The CNAs occurred independently in both BCC and breast cancer and none of them was present in the normal tissue studied. The putative ring or supernumerary marker in the BCC sample (6p21.3 gain) contains the histone cluster 1 H1d gene family, whose super dosage may have affected the modeling of local chromatin with wide consequences on deregulation of genes activity. In addition, the duplication of great number of genes encoding several classes of the MHC antigens may have contributed to the BCC genesis. Regarding the complex genomic pattern of the breast tumor, haplo insufficiency of suppressor genes could play a role in the development and progression of this disease.

## Consent

This study was approved by the institutional ethics committee of the Moinhos de Vento Hospital, Porto Alegre, RS, Brazil (Protocol number 374.454, August 21^st^. 2013), and the patient has given an informed consent for the research and publication of this study and any accompanying images. DNA samples were obtained from patient’s biopsies from normal breast tissue, breast tumor and BCC.

## Electronic supplementary material

Additional file 1: Figure S1: Array-CGH profile of the chromosome 6 from the BCC genome (Agilent 180 K microarray; Genomic Workbench software). The Y axis corresponds to log_2_ values of the ratios between sample (Cy3) and reference DNA (Cy5). A pericentromeric gain at 6p23–q12 can be easily visualized in the chromosome 6 ideogram (blue bars), ranging from chr6: 14838756–68055170 (Genome Build Hg18). This pericentromeric pattern of genomic gain suggested a rearrangement structure of a ring chromosome. (DOCX 138 KB)

Additional file 2: Table S1: Genes involved in DNA loss in breast cancer and gain in BCC samples from the patient studied. Deletion of some gene families could be particularly involved in the development of the breast cancer through haplo insuficiency, as zinc fingers transcription factors (*ZNF*), ubiquitin specific peptidases (*USP),* breast cancer 1A-complex subunit Abraxas *(FAM), besides o*ther important genes, as *CDKN2AIP*, *CASP3*, *TP53*, *BCL2L13*, *BID, NFKB1,* and *TIMP3.* In the BCC, a segment of genes present in the 6p23-p11 is repeated at the 6p21.2-p11.2. Some gene families particularly involved in the amplification, as histones (*HIST*) and major histocompatibility antigens (*HLA*), were identified as well as other genes located in the affected area, as *CDKN1A* and *VEGFA*. (XLSX 27 KB)

Additional file 3: Figure S2: Array-CGH profiles from the breast cancer genome showing CNAs (Agilent 180 K microarray; Genomic Workbench software). The Y axis corresponds to log_2_ values of the ratios between sample (Cy3) and reference DNA (Cy5). **a.** Array-CGH profile of the chromosome 4: the dark gray bars point to the 4p and 4q genomic segments that exhibited losses (.jpg 118 K). **b.** Array-CGH profile of the chromosome 6: the dark gray bars point to 6q genomic regions that exhibited losses (.jpg 118 K). **c.** Array-CGH profile of the chromosome 17: the dark gray bar indicates a deleted area of 17p (.jpg 111 K). **d.** Array-CGH profile of the chromosome 22 showing a whole chromosome aneuploidy (loss) (.jpg 100 K). **e.** A focal ~422 kb micro deletion at 13q13.3 is detailed (shaded dark gray bar); below are indicated the RefSeq genes mapped in the affected genomic region (.jpg 87 K). (DOCX 552 KB)
