# Supplementary figures and images for: Clinical and genetic characterization of basal cell carcinoma and breast cancer in a single patient
Source: Springerplus. 2014 Aug 22;3:454. doi: 10.1186/2193-1801-3-454 (PMC4149681; doi:10.1186/2193-1801-3-454)

Fig. S1


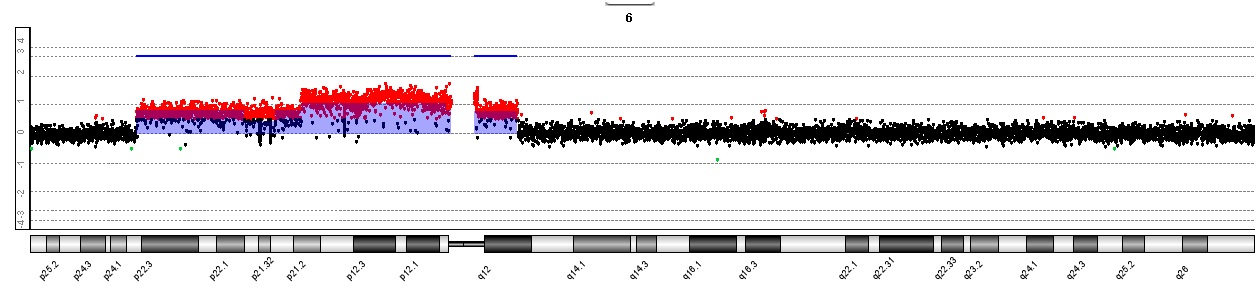

Supplement: Supplementary file 1 — Additional file 1: Figure S1: Array-CGH profile of the chromosome 6 from the BCC genome (Agilent 180 K microarray; Genomic Workbench software). The Y axis corresponds to log2 values of the ratios between sample (Cy3) and reference DNA (Cy5). A pericentromeric gain at 6p23–q12 can be easily visualized in the chromosome 6 ideogram (blue bars), ranging from chr6: 14838756–68055170 (Genome Build Hg18). This pericentromeric pattern of genomic gain suggested a rearrangement structure of a ring chromosome. (DOCX 138 KB) [file 40064_2014_1156_MOESM1_ESM.docx]

**Fig. S2**

1. **Chromosome 4**


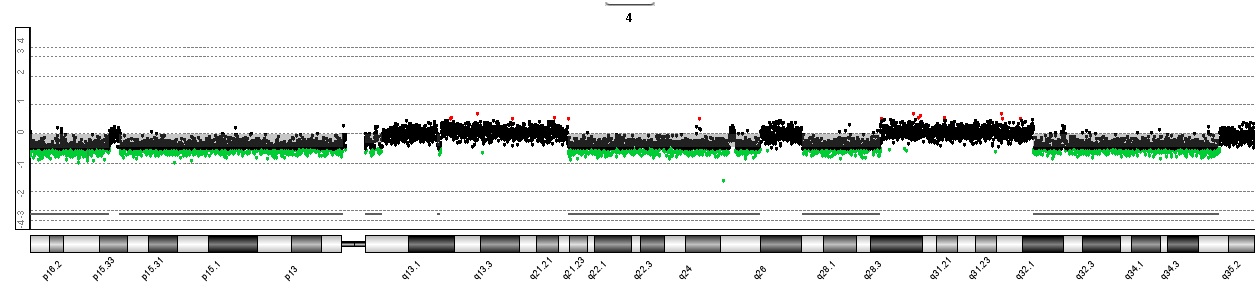


1. **Chromosome 6**


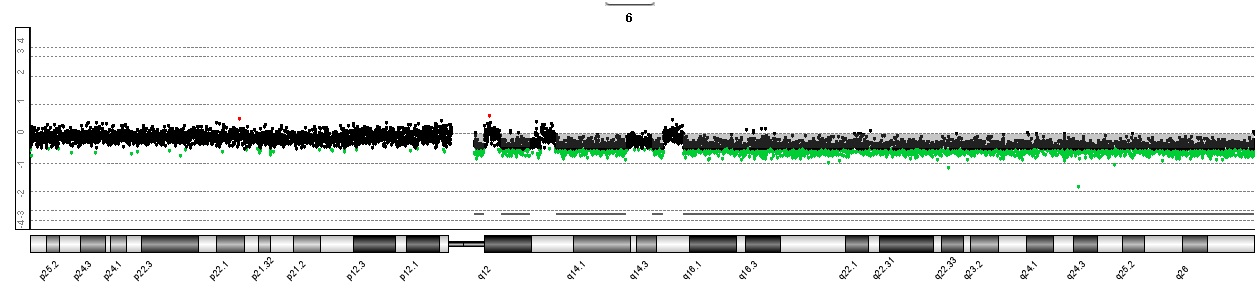


1. **Chromosome 17**


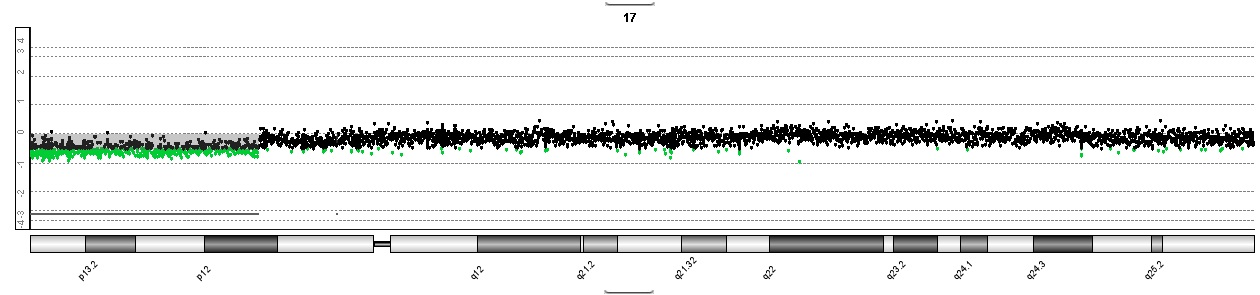


1. **Chromosome 22**


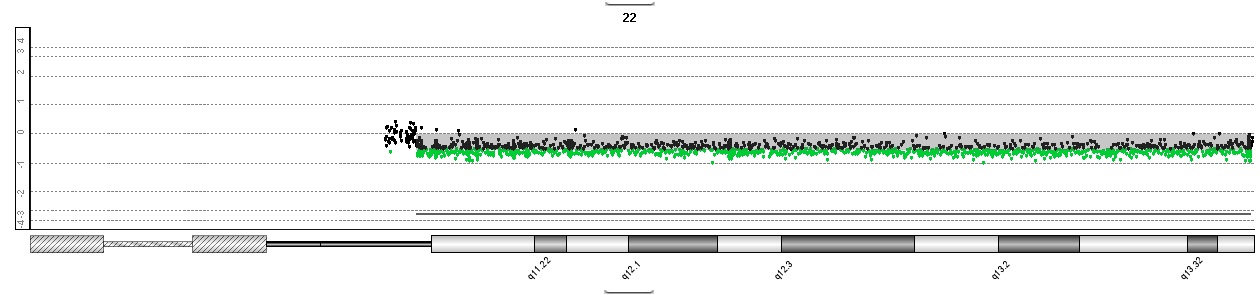


1. **Chromosome 13**


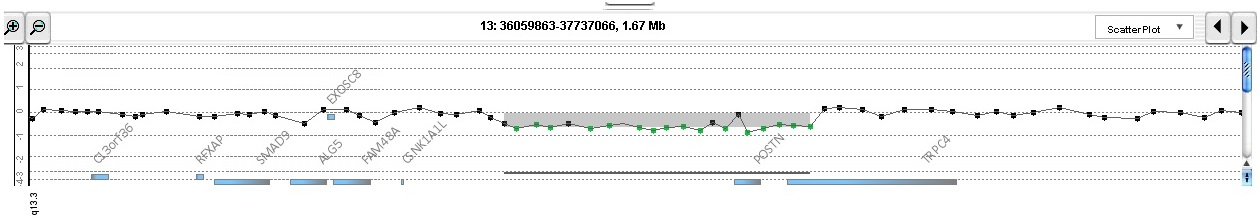

Supplement: Supplementary file 3 — Additional file 3: Figure S2: Array-CGH profiles from the breast cancer genome showing CNAs (Agilent 180 K microarray; Genomic Workbench software). The Y axis corresponds to log2 values of the ratios between sample (Cy3) and reference DNA (Cy5). a. Array-CGH profile of the chromosome 4: the dark gray bars point to the 4p and 4q genomic segments that exhibited losses (.jpg 118 K). b. Array-CGH profile of the chromosome 6: the dark gray bars point to 6q genomic regions that exhibited losses (.jpg 118 K). c. Array-CGH profile of the chromosome 17: the dark gray bar indicates a deleted area of 17p (.jpg 111 K). d. Array-CGH profile of the chromosome 22 showing a whole chromosome aneuploidy (loss) (.jpg 100 K). e. A focal ~422 kb micro deletion at 13q13.3 is detailed (shaded dark gray bar); below are indicated the RefSeq genes mapped in the affected genomic region (.jpg 87 K). (DOCX 552 KB) [file 40064_2014_1156_MOESM3_ESM.docx]
